# Ultrasound versus MRI for evaluation of silicone leakage from silicone breast implants

**DOI:** 10.1016/j.heliyon.2024.e33325

**Published:** 2024-06-19

**Authors:** Karlinde A. Spit, Siham Azahaf, Christel J.M. de Blok, Katya M. Duvivier, Oliver T. Wiebenga, Prabath W.B. Nanayakkara

**Affiliations:** a Section General Internal Medicine, Department of Internal Medicine, Amsterdam Public Health Research Institute, Amsterdam University Medical Centres, location VUmc, Amsterdam, the Netherlands; b Department of Radiology and Nuclear Medicine, Amsterdam University Medical Centres, location VUmc, Amsterdam, the Netherlands

**Keywords:** Silicone breast implants, Ultrasound, MRI, Implant rupture, Silicone depositions

## Abstract

**Background:**

Implant ruptures and gel bleed are not uncommon among women with silicone breast implants. While magnetic resonance imaging (MRI) is traditionally considered the gold standard diagnostic modality, recent studies suggest ultrasound might be an acceptable alternative. This study compares the efficacy of ultrasound and MRI in assessing implant integrity.

**Methods:**

Women with silicone breast implants underwent a breast and axillary ultrasound and MRI on the same day. All tests were assessed by experienced radiologists. The accuracy, sensitivity, and specificity of ultrasound and MRI for implant rupture detection and silicone depositions in axillary lymph nodes were evaluated.

**Findings:**

A total of 104 women participated in the study. The accuracy, sensitivity, and specificity of ultrasound for detecting implant ruptures compared to MRI were 96 %, 95 %, and 96 %, respectively. MRI demonstrated significantly lower sensitivity (44 %) for detecting silicone depositions in axillary lymph nodes compared to ultrasound. A significant association was observed between the presence of enlarged axillary lymph nodes and/or axillary pain and the detection of silicone depositions in axillary lymph nodes on ultrasound (χ^2^ (1, *N* = 104) = 5·1, *p* = 0·024). Six women exhibited silicone depositions in axillary lymph nodes despite having intact first-pair implants, indicative of gel bleed.

**Interpretation:**

Ultrasound is nearly as effective as MRI for detecting breast implant ruptures and is superior for detecting silicone depositions in axillary lymph nodes. We therefore recommend initiating radiological examination in women with breast implants with a breast and axillary ultrasound, proceeding to MRI only if the ultrasound is inconclusive. The prevalence of gel bleed is understudied and its potential adverse health effects might be underestimated. Further research is needed to explore its potential association with development of systemic symptoms.

## 1 Introduction

Breast augmentation surgery using silicone breast implants is the most commonly performed aesthetic plastic surgery procedure worldwide. Almost 2 million breast augmentation surgeries are performed annually worldwide, a number that is only rising [1]. While most women probably do not develop health complaints, local complications are not uncommon and include capsular contraction, breast pain, and implant ruptures. Most implant ruptures are intracapsular (around three-fourths) and the remaining one-fourth are extracapsular [2]. Notably, the 10-year rupture rate of implants is between 10 and 15 % after breast augmentation and between 20 and 25 % after breast reconstruction [[2], [3], [4], [5], [6], [7], [8]].

Implant ruptures are deemed clinically significant due to the recommended removal of affected implants, with the US Food and Drug Administration (FDA) cautioning that removing silicone gel after an implant rupture can be challenging [9]. However, the phenomenon of ‘gel bleed’, wherein silicone particles migrate from implants into the body, remains insufficiently studied [10]. Gel bleed is most commonly demonstrated by presence of silicone depositions in axillary lymph nodes, as silicone particles are thought to spread mainly through the lymphatic system. It is assumed that, even in the absence of an implant rupture, gel bleed can occur in substantial amounts [11].

Mammography, ultrasound, and MRI are the most commonly used breast imaging options. MRI, with its high sensitivity and specificity, is currently considered to be the gold standard for the diagnostic assessment of silicone breast implants. Nonetheless, MRI is costly, has limited availability, and is not suitable for patients with claustrophobia. Additionally, ultrasound is commonly used for the assessment of breast implants. However, the sensitivity and specificity of ultrasound for implant rupture assessments can vary substantially as they are dependent on the experience of the radiologist [12]. Importantly, many radiologists clinically acknowledge ultrasound to be a good modality for assessment of silicone depositions in axillary lymph nodes. One study, with histopathological confirmation of silicone-containing lymph nodes as the gold standard, found that the sensitivity of ultrasound for detecting silicone depositions in axillary lymph nodes was substantially higher than MRI (87·5 % and 20 %, respectively) [13]. Nevertheless, research on this topic is surprisingly scarce as the presence of silicone depositions in axillary lymph nodes is often deemed clinically insignificant.

However, there is a subset of women with silicone breast implants who report systemic symptoms which they attribute to their breast implants. This is referred to as Breast Implant Illness (BII). BII encompasses a variety of symptoms including extreme fatigue, myalgia, arthralgia, morning stiffness, night sweats, and cognitive symptoms such as memory loss [14]. Although the exact pathophysiology of BII is unknown, it is hypothesized that the symptoms are caused by an immune response to the leakage of silicone particles. In this regard, presence of silicone depositions as an indicator of this leakage could be clinically relevant.

More than a decade ago, a specialized silicone outpatient clinic was founded at the Amsterdam University Medical Center, the Netherlands, to assess women with suspected silicone-associated health complaints. A recently published cohort study from this clinic observed that 75 % of women with a suspicion of BII also reported local symptoms [15]. These include stabbing pain and severe itch deep in the breasts and axillaries, often accompanied by axillary lymphadenopathy. The prevalence of local symptoms in this study population decreased significantly from 75 % to 34 % after surgical removal of the implants. In addition, the systemic symptoms the women experienced subsided in 2 out of 3 women after surgical removal of the implants [15]. Based on these clinical findings and previous studies, local and systemic symptoms seem to be associated. Given that gel bleed may be associated with development of systemic symptoms, early and simple detection of silicone leakage may be essential in diagnosing BII in women with breast implants [16].

Therefore, in the current study, we aim to investigate the most effective imaging technique for assessing implant integrity in this patient population, specifically focusing on implant ruptures and signs of gel bleed through presence of silicone depositions in axillary lymph nodes.

## 2 Methods

Women who visited the specialized silicone outpatient clinic of Amsterdam UMC, location VUmc, with suspected BII in 2021–2022 were eligible for inclusion. All radiological tests were performed at Amsterdam UMC. Women who had undergone radiological implant assessment at another clinic during the previous year were not included in this study.

Patients were notified at presentation that their clinical information may be used for research purposes with the aim of improving care and developing a national care pathway for these patients and verbal informed consent was obtained. The study was reviewed by the ethical review board of the Amsterdam UMC, VU University Medical Center Amsterdam (reference number: 2022.0617). It was determined that the Medical Research Involving Human Subjects Act did not apply to this study, and the necessity for written informed consent was waived. The Netherlands Society of Internal Medicine, Netherlands Society of Plastic Surgeons, and the patient-associations were closely involved during the inception and the continuation of this out-patient clinic.

**Fig. 1:**
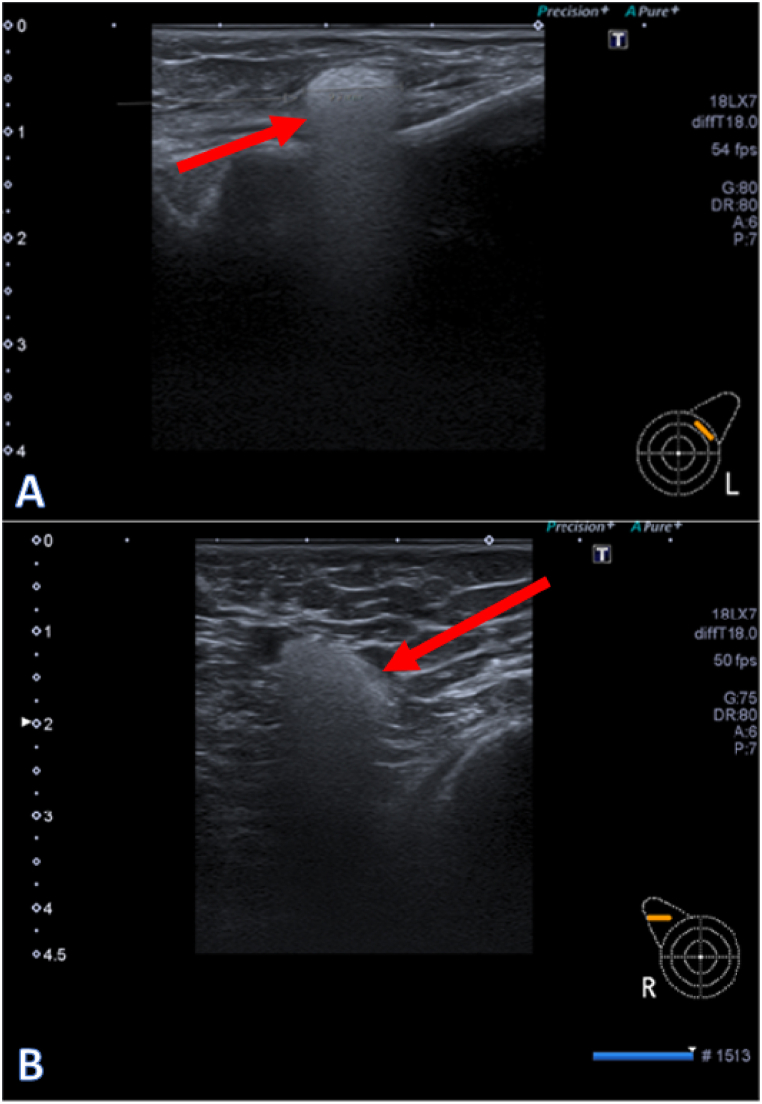
Ultrasound of silicone depositions. **A.**Ultrasound of the left breast showing a silicone deposition. **B.** Ultrasound showing a lymph node with evident snowstorm sign in the right axilla, indicating silicone depositions.

A breast MRI and ultrasound were preferably performed on the same day. The ultrasound of both implants (if applicable) and bilateral axillary lymph nodes were conducted with special interest in the so-called ‘snowstorm phenomenon’ in the breast and axilla, indicating silicone depositions in the lymph nodes (Fig. 1A and B). This snowstorm phenomenon has been validated by multiple studies with histopathological confirmation [13,[17], [18], [19]]. Given the observed high sensitivity and specificity of ultrasound in these studies for silicone detection in axillary lymph nodes, we regarded ultrasound as the gold standard in the current study for the assessment of silicone depositions in axillary lymph nodes. In addition, the implants were accessed by ultrasound for integrity and inhomogeneity and reported as intact, intracapsular rupture, extracapsular rupture, or suspicion of a rupture. As MRI is considered the gold standard for the assessment of implant ruptures, all ultrasounds were performed before the MRI was evaluated. While the MRI and ultrasound were not necessarily evaluated by the same radiologists, all radiological tests were conducted by radiologists specialized in breast radiology. The breast MRI protocol consists of a 3 d T2 Spair series with a sagittal reconstruction, a silicone suppressed series (silicone low), and a water suppressed series (silicone high). According to the breast silicone protocol, the integrity of the implants was scored as intact, internal degradation (water droplets), an intracapsular rupture (keyhole, teardrop, capsular sign, and linguini sign), or an extracapsular rupture [20]. The ultrasound and MRI images were evaluated independently. To increase the accuracy of the results, all ultrasound images with a suspicion of silicone depositions in axillary lymph nodes were reviewed a second time by one experienced radiologist for confirmation.

Statistical analyses were performed using IBM SPSS Statistics (version 28·0; IBM Corp., Armonk, N·Y.). Spearman correlations and chi-square tests were used for comparison of data. Data are shown as mean [SD] in case of normally distributed data and as median [IQR] in case of non-normally distributed data. The sensitivity and specificity of detecting implant ruptures and silicone depositions in axillary lymph nodes with ultrasound and MRI were calculated. Therefrom, the accuracy was calculated, representing the proportion of true positive results (both true positive and true negative) in the selected population. A p-value of <0·05 was considered statistically significant.

## 3 Results

In total, 105 women underwent radiologic assessment with MRI and ultrasound. After second analysis, one woman was excluded for not meeting the study criteria since the MRI images were evaluated before the ultrasound images. Baseline characteristics of the 104 remaining women are shown in Table 1.

**Table 1:** Baseline characteristics.

*Total*	N = 104
*Age in years*	47·3 (±11·8)
*Pair of implants*	
1st pair of implants	72 (69 %)
2nd pair of implants	21 (20 %)
3rd pair of implants	8 (8 %)
≥4th pair of implants	3 (3 %)
*Implant brand*	
Known implant brand (i.e., Allergan, Eurosilicone, Mentor)	70 (67 %)
Unknown brand	34 (33 %)
*Reason for implants*	
Cosmetic	94 (90 %)
Reconstructive	10 (10 %)
*Age of current implants in years*	13·0 [8·0–17·0]
*Total implantation duration in years*	16·0 [10·3–23·8]
*Reported history of ruptured implants*	
Yes	12 (11 %)
No	86 (83 %)
Unknown	6 (6 %)

Data are presented as number (percentage), median [IQR] or mean (±SD).

Most women came to the clinic with their first pair of implants (69 %), with a median implantation time of thirteen years. The majority had breast implants because of cosmetic reasons (90 %). Twelve women were certain they had had ruptured implants in the past, while six women were unsure whether their previous implants had ruptured or not. Almost two-thirds of women reported their implant brand (67 %), while 34 women did not know from which brand their implants were (33 %). Three women presented with a single breast implant and 101 women presented with bilateral implantation, resulting in a total of 205 assessed implants. Local symptoms were reported by 69 women (66 %), consisting of breast pain or intense itching of the breasts, capsular contraction, or pain in the armpits.

### 3.1 Implant ruptures

**Fig. 2:**
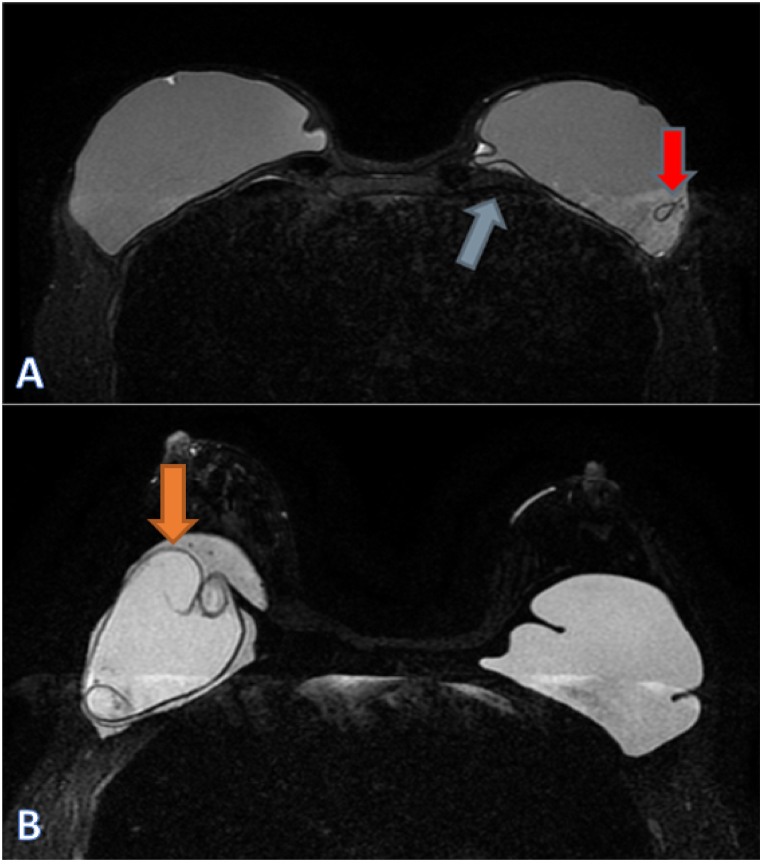
Breast MRI’s with silicone breast implants. **A.** Blue arrow shows a subscapular line, the red arrow points to a teardrop sign. Both are indicative of an intracapsular implant rupture. **B.**Axial MRI silicone suppressed image showing an inhomogeneous implant and internal degradation with linguini sign of the left breast (orange arrow), confirming an intracapsular rupture.

In total, 20 women showed one or two ruptured implants on radiological imaging (Fig. 2A and B). Of the 103 right-sided implants, 13 showed a rupture on MRI, which were all also detected by ultrasound. Of the 102 left-sided implants, twelve showed a rupture on MRI. Of these, eleven ruptures were also detected by ultrasound. The radiologist had a suspicion of a rupture of nine breast implants in eight women (one bilateral) during echography, which could not be confirmed by MRI. This resulted in a high specificity (95 %) and sensitivity (96 %) of ultrasound for the detection of implant rupture, in comparison to the gold standard MRI (Table 2). In addition, accuracy was equally high (95 %). The duration of implantation of the current implants was correlated with the presence of an implant rupture (*r*_*s*_ = 0·257, *p* = 0·009). No correlation was found between implant ruptures and the presence of local symptoms, such as breast pain (*r*_*s*_ = −0·106, *p* = 0·287).

**Table 2:** Sensitivity, specificity, and accuracy of ultrasound in comparison to gold standard MRI for the detection of implant ruptures.

	MRI		
***US***	Implant rupture +	Implant rupture -	
*Implant rupture +*	25	9	
*Implant rupture -*	1	170	
	Sensitivity 96 % (80–100 %)	Specificity 95 % (91–98 %)	Accuracy 95 % (91–98 %)

Data are presented in percentages with 95 % confidence intervals. US: ultrasound. MRI: magnetic resonance imaging.

### 3.2 Axillary silicone depositions

In addition, we compared the axillary lymph nodes on ultrasound and MRI. As previously stated, ultrasound is considered the primary modality for the detection of silicone depositions in axillary lymph nodes. MRI indeed demonstrated a lower sensitivity compared to ultrasound for the detection of silicone depositions in the axillary lymph nodes, as shown in Table 3 (sensitivity 44 %, 95 % CI 25–65 %). No silicone depositions were seen on MRI that were not detected by ultrasound (specificity 100 %, 95 % CI 98–100 %). Fig. 1 shows examples of the typical snowstorm sign. Additionally, the MRI showed enlarged axillary lymph nodes but without a silicone signal in twenty women. In six of them, the ultrasound revealed an evident snowstorm sign, thus indicating silicone depositions. A significant association was observed between presence of enlarged axillary lymph nodes, pain, or intense itching in the armpits and the detection of axillary silicone depositions on ultrasound (*χ*^2^ (1, *N* = 104) = 5·1, *p* = 0.024).

**Table 3:** Sensitivity, specificity, and accuracy of MRI in comparison to ultrasound for the detection of silicone depositions in axillary lymph nodes.

	US		
***MRI***	Axillary silicone +	Axillary silicone –	
*Axillary silicone +*	12	0	
*Axillary silicone -*	15	178	
	Sensitivity 44 % (25–65 %)	Specificity 100 % (98–100 %)	Accuracy 93 % (88–96 %)

Data are presented in percentages with 95 % confidence intervals. US: ultrasound. MRI: magnetic resonance imaging.

### 3.3 Gel bleed

In six women who had their first pair of implants and showed no signs of implant rupture, ultrasound detected silicone depositions in their axillary lymph nodes. This was confirmed by an evident silicone signal on MRI in the same axillary lymph nodes in five out of six women. The duration of their implant placement ranged from 8 to 22 years, with an average of 15.3 years. Subsequently, five out of six women had their implants surgically removed and reported that the shells of all explanted breast implants appeared intact postoperatively.

## 4 Discussion

This study compared the use of ultrasound to MRI for the detection of implant ruptures and silicone depositions in axillary lymph nodes among women with silicone breast implants. We observed a high sensitivity, specificity, and accuracy of ultrasound for the detection of implant ruptures in comparison to gold standard MRI. In addition, ultrasound was superior to MRI for the detection of silicone depositions in axillary lymph nodes. We found an association between the presence of enlarged axillary lymph nodes or axillary pain and the detection of silicone depositions in axillary lymph nodes on ultrasound. Lastly, we observed six women with a first pair of implants without signs of rupture who showed evident signs of silicone depositions in axillary lymph nodes, indicative of gel bleed from the implants.

While the number of breast augmentation surgeries worldwide is increasing, there is currently no consensus regarding the management of systemic complications associated with silicone breast implants. The FDA recommends the first periodic radiologic implant screening 5–6 years postoperatively, and every 2–3 years thereafter [21]. However, adherence to these recommendations is low, with one US study reporting only 5·9 % compliance among their study population [22]. Recently, the FDA updated its guidelines, now suggesting ultrasound as an acceptable alternative for screening asymptomatic patients [23]. Nevertheless, MRI is considered as the gold standard for breast implant assessment in most other countries. The high sensitivity and specificity of gold standard MRI for establishing implant ruptures have been described in multiple earlier studies [[24], [25], [26]]. In our current study, in comparison to MRI, ultrasound showed only one missed rupture and there was a suspicion of a rupture in nine implants that was not confirmed by MRI. Based on these results, we recommend to initially perform an ultrasound in women with breast implants for the detection of implant ruptures and to only consider performing a MRI if the ultrasound is inconclusive. Importantly, performing an ultrasound instead of the MRI is more cost-effective and often easier available, making it accessible for wider adoption in clinical practice.

For decades, some women with breast implants have consequently reported development of systemic symptoms [15,[27], [28], [29]]. The exact pathophysiology remains unclear; however, an immune response to gel bleed has been proposed to play a role [10]. Surprisingly little research has been conducted into the occurrence of gel bleed or the supposed mechanism behind it. Currently, there are no well-defined criteria for establishing gel bleed and we found no studies that investigated the prevalence of gel bleed in women with breast implants. Most likely, this is because gel bleed or even implant ruptures are generally not considered health risks. However, given the consistent reports of women with breast implants and unexplained systemic symptoms, this assumption is at least questionable and deserves more attention in scientific research. Knowledge about potential health risks of silicone leakage through either implant ruptures or gel bleed is currently lacking. This calls for more research into the quantification and possible adverse effects of silicone leakage in the body.

We noticed silicone depositions in axillary lymph nodes with ultrasound as a sign of gel bleed in six women with their first pair of intact implants. In five of these women, the silicone depositions were also seen on MRI images with a silicone signal in the corresponding lymph nodes. Of the five women who underwent implant removal afterwards, none reported an implant shell rupture postoperatively. This supports the hypothesis that accumulation of silicone in lymph nodes can occur in the absence of an implant rupture [11]. While it is often assumed that gel bleed is caused by the silicone gel bleeding through the shell, several observations indicate it might actually be the shell itself that deteriorates. One case report describes a woman with saline breast implants and histopathologically proven silicone depositions in cervical lymph nodes, suggesting these were derived from the silicone shell of the saline implants [30]. Another case report describes a woman with sarcoidosis, in which histopathology with a Laser Raman microprobe analysis revealed the axillary lymph nodes contained silicone structures that are exclusively found in the shell of the implants [31]. These observations support the hypothesis that shell deterioration at least contributes to the accumulation of silicone depositions at distant sites. The prevalence of gel bleed is understudied, most likely underreported and its potential adverse health effects might therefore be underestimated. Given the consistent local and systemic symptoms that some women with breast implants report, further research is needed to investigate a possible association between gel bleed and development of systemic symptoms. In light of the above, the difference in sensitivity for the detection of silicone depositions in axillary lymph nodes on MRI and ultrasound should not be underestimated. Especially in women with silicone breast implants who report local symptoms such as breast or axillary pain or axillary lumps, gel bleed might be indicative of an association with the implants. In this patient group, ultrasound should be the modality of choice for the evaluation of implant status. In particular, the axillary lymph nodes should be part of the standard assessment.

We believe our study contributes to optimizing the clinical care pathway for women with silicone breast implants who report local and systemic symptoms. In addition, it contributes to the limited knowledge on gel bleed and might stimulate more research into its potential adverse health effects. However, this study has several limitations that need to be addressed. The relatively small sample size and selection of patients used for this study (i.e., women with breast implants who report systemic symptoms) may limit our ability to extend our results to the general population of all women with breast implants. Secondly, all ultrasounds were performed by experienced radiologists who specialize in breast diagnostics. The high sensitivity and specificity of ultrasound observed in this study might not be applicable to all radiologists in general.

In conclusion, our results indicate ultrasound is an effective, cheap, and accessible diagnostic modality for evaluating silicone breast implant status. In addition, ultrasound proves to be the modality of choice for diagnosing silicone depositions in axillary lymph nodes as an indication of gel bleed. Therefore, we recommend initiating the radiological examination of breast implants with an ultrasound of both breasts and axillae, and only performing a MRI if the ultrasound results are inconclusive. We highlight the importance of further research into the possible association between gel bleed and development of systemic symptoms in women with silicone breast implants.

## Data availability statement

Data will be made available on request.

## Funding

This research was financially supported by the research program on silicone breast implants, coordinated by the National Institute of Public Health and the Environment on behalf of the Ministry of Health, Welfare, and Sport of the Netherlands.

## CRediT authorship contribution statement

**Karlinde A. Spit:** Writing – review & editing, Writing – original draft, Project administration, Methodology, Investigation, Formal analysis, Data curation, Conceptualization. **Siham Azahaf:** Writing – review & editing, Validation, Methodology. **Christel J.M. de Blok:** Writing – review & editing, Supervision, Methodology. **Katya M. Duvivier:** Writing – review & editing, Investigation, Data curation, Conceptualization. **Oliver T. Wiebenga:** Writing – review & editing, Investigation, Data curation, Conceptualization. **Prabath W.B. Nanayakkara:** Writing – review & editing, Supervision, Project administration, Conceptualization.

## Declaration of competing interest

The authors declare that they have no known competing financial interests or personal relationships that could have appeared to influence the work reported in this paper.
